# Internet addiction in light of social connectedness and connectedness to nature

**DOI:** 10.1192/j.eurpsy.2022.1526

**Published:** 2022-09-01

**Authors:** M. Gawrych

**Affiliations:** The Maria Grzegorzewska University, Institute Of Psychology, Warsaw, Poland

**Keywords:** connectedness, internet addiction, mental health

## Abstract

**Introduction:**

The Internet increasingly influences the lives of people in pandemic times. Although there are many positives, there are also risks related to excessive use and addiction. Internet addiction subject has been explored worldwide.

**Objectives:**

The aim of this pilot study was to analyze the relationships between social connectedenss, connectedness to nature and the occurrence of Internet addiction.

**Methods:**

The data were collected from a group of 200 young adults. A cross-sectional observational study using an online questionnaire was conducted via social media. The semi-structured online questionnaire covered the following areas: (1) general sociodemografic data; (2) Internet usage, measured by Generalized and Problematic Internet Use Scale (GPIUS2) (Caplan, 2002), Internet Gaming Disorder Scale–Short-Form (IGDS-SF9) (Pontes & Griffiths, 2015), the Bergen Facebook Addiction Scale (BFAS) (Andreassen et al., 2012); (3) nature conectedness, measured by the Connectedness to Nature Scale (CNS) (Mayer, Frantz, 2004); (4) social connectedness, measured by the Social Connectedness Scale Revised (SCS) (Lee et al., 2001); (4) psychological impact and mental health, measured by Depression, Anxiety, Stress Scale (DASS-21) and (5) psychological features, such as coping strategies (Mini-COPE, Carver et al.,1989) and personality traits (TIPI -Gosling, Rentfrow, Swann Jr., 2003)

**Results:**

The detailed results and key findings will be presented during the congress.

**Conclusions:**

As the research of the desribed area is insufficient so far, this pilot study may provide a significant contribution to the knowledge on new aspects of internet addictions’ mechanisms. Moreover, it is predicted that our result may have scientific influence on both research in connectedness and eco-psychology.

**Disclosure:**

No significant relationships.

